# Contemporary Challenges and Strategies for Diagnosing and Managing in Type 2 Myocardial Infarction: A Narrative Review

**DOI:** 10.3390/jpm16080438

**Published:** 2026-08-21

**Authors:** Julia Kościanek, Natalia Zabawa, Anna Stanaszek, Michał Maślanka, Hubert Mączka, Martyna Pniaczek, Aleksandra Bołoz, Jadwiga Nessler, Jarosław Zalewski, Konrad Stępień

**Affiliations:** 1 Student Research Group, Department of Coronary Artery Disease and Heart Failure, Jagiellonian University Medical College, 31-008 Kraków, Poland; 2 Department of Coronary Artery Disease and Heart Failure, St. John Paul II Hospital, 31-202 Kraków, Poland; 3 Department of Coronary Artery Disease and Heart Failure, Institute of Cardiology, Jagiellonian University Medical College, 31-008 Kraków, Poland; 4 Department of Thromboembolic Disorders, Institute of Cardiology, Jagiellonian University Medical College, 31-008 Kraków, Poland

**Keywords:** type 2 myocardial infarction, individualized clinical decision-making, clinical phenotypes

## Abstract

Type 2 myocardial infarction (T2MI) occurs secondary to an imbalance between myocardial oxygen supply and demand, with systemic conditions serving as the primary precipitating factors. T2MI predominantly affects older adults and is frequently accompanied by multiple chronic comorbidities. Therefore, it often has an atypical clinical course and remains a significant diagnostic and therapeutic challenge, particularly given the lack of standardized clinical guidelines. Furthermore, the diagnosis of T2MI in women is especially challenging due to sex-related differences in biomarker kinetics and atypical symptom presentation. The aim of this review is to summarize current knowledge on T2MI, discuss modern diagnostic tools, and analyze sex-related differences in this context in order to identify future directions for the development of therapeutic guidelines. The review emphasizes the need for a multimodal approach, integrating biomarkers, individualized clinical decision-making and advanced cardiac imaging, as differentiation between T2MI and T1MI remains a significant challenge. Therefore, both non-invasive and invasive diagnostic strategies may be required to accurately identify patients with T2MI. Although routine coronary angiography in T2MI is not clearly supported by current evidence, it may be useful as a decisive tool for reclassification, particularly when the clinical presentation is ambiguous.

## 1. Introduction

Myocardial infarction (MI) is a heterogeneous entity that may be classified into several subtypes based on pathological, clinical, and prognostic differences, implying different treatment strategies. Type 2 myocardial infarction (T2MI) is defined as an ischemic injury driven by a mismatch between oxygen supply and demand, with the concurrent exclusion of acute atherothrombotic plaque disruption [1,2,3]. Unlike Type 1 MI (T1MI), T2MI is often triggered by secondary clinical stressors such as tachyarrhythmia, bradyarrhythmia, severe anemia, hypertension, coronary artery spasm or respiratory failure. Furthermore, epidemiological data suggest a higher proportion of females among patients with T2MI compared to T1MI (36% vs. 26%, *p* = 0.002) [1,4]. The clinical identification of this entity continues to be challenging, mainly due to the lack of a single, pathognomonic feature. High-sensitivity cardiac troponin (hs-cTn) plays a crucial role in diagnosis, but its interpretation requires caution, especially in the female population. Due to acute vessel occlusion in T1MI, the troponin rise is rapid, steep, and consistent with extensive necrosis, whereas T2MI typically presents with a blunted rise-and-fall pattern, and more modest elevations, reflecting a situation of supply–demand mismatch [1,5,6].

The electrocardiogram (ECG) classification of ST-segment elevation myocardial infarction (STEMI) and non-STEMI (NSTEMI) is applicable to both types of MI. However, ST-segment elevation occurs less frequently in T2MI, with a reported incidence of 1% to 24% [7]. Patients with T2MI are also less likely to demonstrate ischemic ECG changes or regional wall motion abnormalities [8,9,10]. Nevertheless, invasive diagnostics in T2MI remain complex. The absence of significant stenoses does not rule out T1MI and advanced coronary artery disease (CAD) is frequently identified in patients with T2MI. Notably, T2MI can occur both in the presence of significant stenoses and in angiographically normal coronary arteries [9,11,12,13,14,15]. The aim of this review is to summarize current knowledge on T2MI, discuss modern diagnostic tools, and analyze sex-based differences in this context to identify future directions for therapeutic guidelines.

## 2. Definition

According to the Fourth Universal Definition of Myocardial Infarction (UDMI) established in 2018, T2MI is currently defined as myocardial necrosis secondary to an imbalance between myocardial oxygen supply and demand, unrelated to acute atherothrombotic coronary plaque rupture or thrombosis [1]. Its diagnosis requires a dynamic rise and/or fall in cardiac troponin values with at least one value exceeding the 99th percentile upper reference limit, along with clinical evidence of acute myocardial ischemia.

Historically, acute myocardial infarction was primarily understood as a consequence of atherothrombotic coronary disruption. The conceptualization of T2MI has evolved considerably over the last two decades, shifting to emphasize a non-thrombotic imbalance between myocardial oxygen supply and demand as its central pathophysiological mechanism. While the formal distinction between T2MI and plaque-rupture events was introduced in the 2007 UDMI and reviewed in 2012, the 2018 update particularly stressed the need to differentiate true T2MI from acute myocardial injury [1,2,3]. The consensus accentuated that elevated troponin concentrations alone are insufficient for an infarction diagnosis, as many critically ill patients experience myocardial injury without overt ischemic features.

Specifically, acute myocardial injury is characterized by elevated cardiac troponin levels without clinical evidence of ischemia, whereas T2MI requires additional ischemic features, such as chest pain, ischemic electrocardiographic changes, imaging evidence of new loss of viable myocardium, or new regional wall motion abnormalities [9,16]. Establishing this distinction is still a clinical challenge and continues to provoke substantial debate in the contemporary cardiology literature. This demarcation carries considerable implications for epidemiology, prognosis, therapeutic strategies, and the establishment of inclusion criteria in clinical studies. Recent investigations have demonstrated marked interobserver variability in adjudicating T2MI, underscoring the need for more objective diagnostic tools.

## 3. Clinical Presentation

The clinical presentation of T2MI tends to diverge from T1MI, as T2MI typically affects older adults, is more common in females, and is associated with multiple chronic comorbidities such as chronic kidney disease, heart failure, chronic obstructive pulmonary disease, anemia, diabetes mellitus, and atrial fibrillation [17,18]. As a result, frailty and polypharmacy are common. In contrast to T1MI, which is usually triggered by an acute atherothrombotic event, T2MI arises secondary to another acute medical or surgical condition that leads to an imbalance in myocardial oxygen supply and demand.

Due to this clinical background, T2MI presents with highly variable and often atypical symptoms [1]. Ischemic chest pain may occur but is often absent, especially in elderly, diabetic, or critically ill patients. Symptoms are usually dominated by the underlying illness or appear as non-specific signs such as acute dyspnea, generalized weakness, altered mental status, or unexplained hypotension [9]. Objective findings are also variable. Electrocardiograms may show ST-segment depression, transient ST-segment elevation, T-wave inversion, non-specific repolarization changes, or may be normal [19]. Although imaging and biomarkers are essential for diagnosis, initial troponin elevations and echocardiographic wall motion abnormalities often overlap with other acute conditions [4].

Because of the clinical overlap and the close association of symptoms with the underlying condition, a structured approach is fundamental for accurate differential diagnosis. The following sections provide a more detailed overview of the diagnostic evaluation needed to distinguish T2MI.

## 4. Etiology and Pathophysiology

A central underlying mechanism in T2MI is a hypoxemic cascade that leads to cytotoxic cellular damage. This process entails mitochondrial dysfunction, oxidative stress, and disruption of calcium homeostasis, ultimately resulting in cardiomyocyte cytolysis [20,21]. At the cellular level, cardiomyocytes appear to respond similarly to those in T1MI. Nonetheless, these mechanisms primarily result in subendocardial ischemia rather than transmural infarction [5]. Importantly, T2MI is often considered to be a more global ischemic phenomenon with regional myocardial dysfunction influenced by the concomitant CAD [21].

Myocardial oxygen supply depends on coronary blood flow and the blood’s oxygen-carrying capacity, whereas its demand is determined mainly by heart rate, myocardial contractility, and systolic wall tension [22]. The imbalance between those two factors is typically triggered by acute stressors that either increase myocardial oxygen requirements or reduce coronary perfusion. Habitually, it is multifactorial and reflects a high burden of comorbidities, which contributes to the clinical complexity and worse prognosis of T2MI.

According to the Fourth UDMI [1], T2MI arises from various pathophysiological mechanisms. These include increased myocardial oxygen demand, as seen in severe hypertension or catecholamine excess, as well as a reduction in oxygen supply in conditions such as anemia, hypoxia, hypotension, tachycardia or bradyarrhythmia. The postoperative setting is particularly high-risk, as patients commonly face multiple triggers such as hemorrhage, catecholamine excess, and hemodynamic instability [23]. These factors markedly increase T2MI incidence and additionally complicate the diagnostic assessment.

Nonetheless, systemic conditions are the primary precipitating factors, with anemia, tachyarrhythmias, and respiratory failure being the most common triggers of T2MI [24]. Anemia impairs oxygen supply to tissues and, particularly in the presence of multiple comorbidities, markedly increases the risk of secondary myocardial infarction. Tachyarrhythmias increase myocardial oxygen demand by augmenting contractile work and shortening diastole, consequently reducing coronary perfusion. Additionally, a reduction in stroke volume may further decrease coronary perfusion pressure, exacerbating myocardial ischemia [24]. Respiratory failure contributes through hypoxia and increased right ventricular afterload due to elevated pulmonary pressure, promoting cardiomyocyte injury or death [25].

In addition to systemic triggers, mechanisms directly related to reduced coronary perfusion—including fixed atherosclerotic disease without plaque rupture, coronary spasm, coronary embolism, or spontaneous coronary artery dissection—may likewise contribute to T2MI [1]. However, de Lemos et al. [26] challenged this classification, stating that these conditions are primary coronary events rather than supply–demand mismatches. They recommended reintegrating such cases into a broader T1MI category or a new ‘vascular’ subcategory, as their management and prognosis are more similar to atherothrombotic events than to systemic illness.

The ischemic threshold varies among individuals, as it depends on the individual’s capacity to maintain adequate coronary perfusion under stress, which is influenced by age, comorbidities, and the extent of underlying CAD. It is important to note that atherosclerotic plaque burden is common in T2MI, with studies reporting that its presence is between 40% and 70% in T2MI patients. However, its true prevalence is likely underestimated, as patients with T2MI less frequently undergo coronary angiography [27]. Notably, the presence of CAD in this population is associated with increased mortality and a higher risk of cardiovascular complications [28].

Recent studies have proposed distinct phenotypic profiles of T2MI based on underlying vascular pathology and clinical presentation [29]. Phenotype 1 T2MI typically occurs in younger patients with fewer comorbidities and a lower burden of atherosclerosis. It is primarily associated with non-atherosclerotic mechanisms that reduce coronary blood flow, such as coronary vasospasm or microvascular dysfunction. In contrast, phenotype 2 T2MI is characterized by more significant CAD and systemic stressors that precipitate an acute mismatch between oxygen supply and demand, most commonly observed in older patients with multiple comorbidities [29].

T2MI occurs more prevalently within the elderly population [30], as advanced age is associated with progressive vascular changes, including arterial stiffening and dilation. These structural changes and increased microvascular resistance significantly reduce coronary reserve and worsen endothelial dysfunction. Aging also impairs the heart’s adaptation to acute stress by decreasing arterial compliance and catecholamine responsiveness [31]. Collectively, age-related physiological changes reduce the threshold for supply–demand imbalance, thereby increasing the susceptibility of older patients to T2MI during episodes of hemodynamic instability.

## 5. Differential Diagnosis

Current data indicate that the changes observed on admission ECG—namely ST-segment elevation, reciprocal changes, and ventricular repolarization abnormalities in the form of ST-segment deviations and T-wave inversions—are associated with a high probability of type T1MI [32,33]. In accordance with current guidelines, patients with STEMI should be referred for invasive coronary angiography without any delay for blood sampling or other diagnostic tests [34,35]. Nonetheless, distinguishing patients with NSTEMI is not straightforward and requires more in-depth diagnostic evaluation.

Moreover, as noted in numerous studies, patients with T2MI are typically older, more often female, and have multiple comorbidities [36,37,38]. However, due to the progressive aging of the population, there is an increasing overlap between the phenotypes of patients with type 1 and type 2 myocardial infarction. An additional challenge is that some of the triggers of T2MI—such as arrhythmias, respiratory failure, and heart failure—are also complications of T1MI [39,40]. Early, non-invasive differentiation between subtypes remains an imposing diagnostic hurdle for many clinicians [35,36,41].

## 6. Multimodal Diagnostic Approach

### 6.1. Noninvasive Diagnostic Modalities

High-sensitivity cardiac troponin (hs-cTn) is currently a widely utilised and well-validated biomarker in the diagnosis of acute MI [1]. However, numerous studies indicate that although differences in hs-cTn concentrations exist between patients with T1MI and T2MI [35,42,43], both types are characterized by levels exceeding the sex-specific upper reference limit (URL) (99th percentile), along with significant dynamic changes [36]. Hs-cTn concentrations at presentation are typically higher in T1MI than in T2MI. However, absolute values vary considerably across assays and patient cohorts. For instance, one study using hs-cTnT reported median concentrations of 63 ng/L in T1MI and 22–28 ng/L in T2MI, depending on the definition applied [44]. In a separate large cohort using hs-cTnI [45], median concentrations were 91 ng/L in T1MI and 50 ng/L in T2MI. Notably, there was substantial overlap in interquartile ranges (30–493 ng/L for T1MI versus 22–147 ng/L for T2MI), which limits the discriminatory value of troponin concentration alone at presentation [45,46,47].

It can thus be concluded that the use of hs-cTn as a standalone test does not permit reliable differentiation between T1MI and T2MI. Consequently, a plethora of studies have investigated the potential of combining biomarkers to enhance the distinction between these two categories of MI. An example of such an approach is the ratio of hs-cTn to NT-proBNP, which has demonstrated potential in distinguishing between subtypes in several studies [48].

NT-proBNP is released in response to myocardial wall stretch and stress, thus acting as a sensitive marker of cardiovascular strain, even at a subclinical stage [49,50,51]. It is noteworthy that the levels of this substance are also elevated in conditions such as renal failure and atrial fibrillation, which occur more frequently among patients with T2MI [37,38]. This finding is consistent with the underlying pathophysiology of this subtype of MI, which is characterised by a myocardial oxygen supply–demand imbalance and structural strain, rather than acute plaque rupture. Consequently, while T1MI is characterised by higher troponin peaks due to acute necrosis, T2MI often presents with significant NT-proBNP elevation, indicating the underlying hemodynamic stress [36,52]. Memon et al. [52] reported that the NT-proBNP-to-troponin ratio was more than 3-fold higher in T2MI cases (94,880 ± 152,648 vs. 24,209 ± 78,727; *p* = 0.007), reflecting differences in the implicit pathophysiological mechanisms. Evidence demonstrates that NT-proBNP, when used in isolation, has been shown to have comparable performance to the NT-proBNP/troponin ratio. However, it is important to note that both of these approaches present only moderate discriminatory ability (area under the curve, AUC: 0.717 and 0.720, respectively) [52]. Nevertheless, the NT-proBNP level is responsive to a wide range of both cardiac and non-cardiac stressors. Consequently, its specificity remains inherently constrained, requiring its consideration alongside other biomarkers and broader clinical assessments.

Additional insights may be derived from the relationship between troponin and creatine kinase-MB (CK-MB). In T2MI, troponin elevation tends to be disproportionately higher relative to CK-MB compared with T1MI, as reflected by a higher peak cTnT/CK-MB ratio (0.09 vs. 0.06, *p* = 0.06) [53].

Among the multiple inflammatory markers, C-reactive protein (CRP) levels are consistently higher in patients with T2MI compared with T1MI, and the CRP/troponin ratio has shown augmented diagnostic accuracy, achieving an AUC of approximately 0.84 with high specificity for T2MI discrimination [54,55,56].

Conversely, apolipoprotein A-II, which is present at lower concentrations in T2MI, represents an additional component of multimarker strategies [54]. The highest diagnostic performance has been achieved using multi-biomarker panels. In particular, the four-biomarker model from the BACC study (apolipoprotein A-II, NT-proBNP, copeptin, hs-cTnI) reached an AUC of 0.82, while the six-biomarker panel derived from the CHOPIN study attained an AUC of 0.85, despite not incorporating hs-cTn assays [54,57,58].

A promising strategy has been proposed by Snavely et al., involving the application of a machine learning algorithm to differentiate between T1MI and T2MI [36]. The diagnostic performance was found to have improved significantly when NT-proBNP and Galectin-3 (Gal-3) were integrated into the MI3 algorithm. It should be noted that the MI3 algorithm traditionally relies on serial hs-cTnI measurements. Embedding these markers into the model resulted in an AUC value of 0.797, which was utilized for the purpose of differentiating between MI subtypes. The addition of Gal-3 is of particular pertinence in this context, as it functions as a pivotal mediator of fibrosis, inflammation, and ventricular remodelling [59]. As emphasized by the authors, these preliminary results imply that a multimarker, machine learning-based approach holds promise; however, larger prospective validation studies are needed.

Other biomarkers of interest present additional perspectives on the complex hemodynamics of T2MI. For instance, MR-proANP (mid-regional pro-atrial natriuretic peptide) functions as a reliable indicator of atrial wall stretch and volume overload [60,61]. Similarly, CT-proET-1 (C-terminal pro-endothelin-1) provides a window into vasoactive regulation, reflecting processes such as myocardial hypertrophy, arrhythmias, and fluid retention [62]. Furthermore, MR-proADM (mid-regional proadrenomedullin) has attracted interestfor its release amid critical states, including ventricular volume overload and sepsis [63,64]. Together, these markers reflect the pathophysiological profile of T2MI, characterized by congestion, vascular stress, and neurohormonal activation, which represent drivers for the supply–demand mismatch [58,65]. Horiuchi et al. [58] reported that the use of the aforementioned biomarkers alone achieved high diagnostic accuracy (AUC = 0.854), comparable to a model incorporating clinical variables and cTnI (AUC = 0.884). It should be noted, however, that the authors stressed the hypothesis-generating nature of their findings, partly due to the relatively small study population, and highlighted the need for onward validation in independent cohorts.

Another biomarker investigated for its aptitude to distinguish between MI subtypes is copeptin, the C-terminal portion of the vasopressin precursor, which acts as a reliable marker of the acute endogenous stress response [66]. Nonetheless, findings across studies evaluating its standalone diagnostic value remain inconsistent [34,57,67], with some reports indicating only low-to-moderate diagnostic performance. For instance, one study reported a low-to-moderate performance with an AUC of 0.66 within the derivation cohort, which dropped to 0.58 in the validation cohort [58]. This absence of discriminatory capability may be attributable to the observation that copeptin levels exhibit a marked increase in a variety of critical conditions, encompassing sepsis and heart failure, which are prevalent among both T1MI and T2MI patients. This phenomenon serves to constrain the specificity of the test, thereby impeding its capacity for differentiating between different types of infarction. However, this diagnostic limitation may be overcome by shifting from a single-marker approach to a multi-marker strategy. Despite the fact that copeptin levels are considerably elevated in T2MI in comparison to the control group (21.4 vs. 8.1 pmol/L), this biomarker only exhibits its complete clinical potential when incorporated into multi-parametric models in conjunction with conventional markers of myocardial injury [39]. Incorporating copeptin into a multi-marker panel allows for a synergistic evaluation of both hemodynamic stress and cardiomyocyte necrosis, additionally enhancing discrimination and successfully increasing the AUC to approximately 0.94 [68]. Nevertheless, within the framework of T2MI, this elevated sensitivity to general ischemia does not directly translate into a high discriminatory capacity for subtype differentiation.

The role of biomarkers in the diagnosis and prognosis of T2MI remains unclear. Therefore, current guidelines still recommend using a detailed medical history, physical examination, and standard laboratory tests for establishing the diagnosis [69]. It is imperative to emphasize that these methodologies are intrinsically subjective and susceptible to interpretive variability. It is evident that further research is required on multi-biomarker panels in order to establish more objective diagnostic criteria.

Electrocardiographic (ECG) findings in T2MI are generally less specific for acute transmural ischemia compared with T1MI. In large observational cohorts, ST-segment elevation and pathological Q waves are significantly less frequent in T2MI (ST elevation: 14.1% vs. 44.2%; OR 0.22 and Q waves: 6.7% vs. 20.8%; OR 0.38), reflecting the lower prevalence of acute transmural ischaemia in this population [33,42]. In contrast, T2MI is more commonly associated with non-specific ST–T wave abnormalities (24.7% vs. 10.8%; OR 2.62) and atrial arrhythmias (21% vs. 6.6%; OR 4.99), consistent with systemic oxygen supply–demand imbalance as the underlying mechanism [19,42,69]. No reproducible differences have been observed in the prevalence of ST-segment depression or T-wave inversion between T1MI and T2MI, limiting their diagnostic specificity [42,69]. Although dynamic ST-segment changes may signal ongoing acute myocardial ischemia and identify patients who could benefit from urgent invasive evaluation, they occur in only a minority of cases and lack sufficient accuracy to reliably differentiate MI subtypes [33]. As emphasized in current ESC guidance, ECG findings must be interpreted in conjunction with clinical context, biomarker dynamics, and imaging modalities to ensure accurate classification of myocardial injury [1].

Transthoracic echocardiography (TTE) is a fundamental first-line tool for rapid assessment of left ventricular function and regional wall motion abnormalities (RWMA). However, its ability to distinguish between MI subtypes is limited. Evidence indicates that TTE is used less often in T2MI patients, but when performed, it often shows RWMA and ejection fraction (EF) patterns similar to those in T1MI [5,42]. Despite the fact that a significant proportion of T2MI patients exhibit preserved EF or unremarkable findings, the employment of advanced techniques, such as Global Longitudinal Strain (GLS), has been demonstrated to improve the sensitivity of uncovering subtle myocardial dysfunction. However, the routine use of GLS for subtype differentiation has not yet been standardised in clinical practice [5].

Coronary computed tomography angiography (CCTA) has emerged as a valuable non-invasive imaging modality in the diagnostic evaluation of T2MI. CCTA enables detailed visualization of coronary anatomy, facilitates the assessment of ischemia-related changes, and permits reliable exclusion or confirmation of obstructive CAD. Systematic coronary assessment in patients with T2MI is warranted due to the high prevalence of atherosclerotic burden in this population [55,70,71]. The presence of CAD is associated with worse prognosis, with 5-year mortality rates reaching 62.5% in T2MI compared with 36.7% in T1MI, and contributes to a lower ischemic threshold, predisposing patients to myocardial injury even under mild physiological stress [6]. Despite this, evidence-based secondary prevention therapies remain underutilized in this population [9]. In studies such as DEFINE TYPE 2 MI and DEMAND-MI, CAD was identified in up to 68–92% of patients, with obstructive disease present in approximately 26–30%, and imaging led to changes in diagnosis in a subset of cases [70,71]. In addition to anatomical assessment, CT-derived fractional flow reserve (FFR-CT) allows evaluation of the hemodynamic significance of coronary lesions and illustrates the multifactorial nature of ischemia in T2MI [70]. CCTA is particularly useful in hemodynamically stable patients without known CAD and with low-to-intermediate pre-test probability, where it enables reliable exclusion of significant stenosis and detection of subclinical atherosclerosis [6,70].

Cardiac magnetic resonance (CMR) is a key noninvasive tool for myocardial tissue characterization in patients with suspected myocardial infarction and persistent diagnostic uncertainty. Using the updated 2018 Lake Louise criteria, CMR allows differentiation between ischemic (subendocardial or transmural late gadolinium enhancement [LGE]) and non-ischemic (mid-wall or epicardial) injury patterns, while the detection of myocardial edema enables distinction between acute and chronic injury [1,72,73]. In patients with suspected T2MI or MINOCA, CMR can identify the underlying etiology in up to 70–87% of cases, including myocarditis, cardiomyopathies, and true ischemic damage. Notably, CMR cannot directly distinguish T1MI from T2MI, as both share identical imaging features of ischemic injury [33,73,74,75,76]. However, certain findings, such as larger infarct size, microvascular obstruction, or intramyocardial hemorrhage, are more suggestive of T1MI, whereas smaller or multi-territorial infarcts may indicate T2MI, although these features are not specific. Stress perfusion CMR may further detect microvascular dysfunction or functionally significant coronary disease [74,77]. Overall, CMR plays a complementary role by confirming ischemic injury and excluding alternative diagnoses, while definitive differentiation requires integration of clinical context, biomarkers, and coronary imaging.

### 6.2. Invasive Diagnostic Strategies

The clinical role and optimal timing of invasive diagnostic and therapeutic interventions in T2MI remain highly debated in contemporary cardiology. Although current guidelines clearly recommend urgent coronary angiography and subsequent revascularization for T1MI, evidence supporting routine invasive strategies in T2MI is limited. As a second subtype arises secondary to a broad range of underlying non-thrombotic conditions, management primarily focuses on addressing the underlying cause and correcting the precipitating condition after patient stabilization. In this context, routine invasive interventions may not provide clinical benefit and may instead expose vulnerable, multimorbid patients to unnecessary procedural risks [11,78]. Accordingly, the literature reports conflicting findings regarding the prevalence of significant coronary artery stenosis and acute plaque rupture among patients initially classified as having T2MI [37,79,80].

Although randomized controlled trials specifically evaluating the impact of invasive strategies on clinical outcomes are lacking, observational and registry data, including the SWEDEHEART and MINAP registries, indicate a potential survival benefit [81,82]. The baseline utilization of invasive strategies in T2MI remains relatively low, ranging from 20% to 60%. However, a meta-regression analysis of 3155 patients found that higher rates of percutaneous coronary intervention (PCI) correlated with reduced 1-year mortality [83]. Similarly, a large U.S. cohort study associated revascularization with lower adjusted in-hospital mortality, without a significant effect on 30-day readmission rates for death, heart failure, or recurrent infarction [84]. This benefit may be partially attributable to the high prevalence of previously unrecognized CAD, identified in up to two-thirds of T2MI patients, often presenting as multivessel and diffuse disease. Identifying significant atherosclerotic burden has important therapeutic implications, as it leads to the initiation or optimization of secondary prevention therapies, such as antiplatelet and lipid-lowering treatments [71]. Nevertheless, since these data are primarily based on observational studies, definitive conclusions regarding the clinical and economic value of an early invasive strategy require results from ongoing randomized clinical trials, such as the ACT-2 trial [85].

In clinical practice, coronary angiography serves as a decisive tool for reclassification, particularly when distinguishing T2MI from T1MI when the clinical picture is ambiguous. Although not routinely required for diagnosing T2MI, angiographic assessment enables direct visualization of the coronary anatomy. This approach is critical for excluding T1MI-defining features, such as plaque disruption or intracoronary thrombus, while simultaneously identifying non-atherothrombotic causes of myocardial injury, including coronary vasospasm, embolism, or spontaneous coronary artery dissection (SCAD) [11,72,86]. The threshold for invasive assessment should be lower in patients with persistent or recurrent ischemia, dynamic electrocardiographic changes, or high-risk clinical features. This consideration is especially important in elderly individuals or those with baseline electrocardiographic abnormalities, where clinical evaluation alone may be insufficient. In addition, intravascular imaging modalities, including optical coherence tomography (OCT) and intravascular ultrasound (IVUS), yield additional mechanistic insights by detecting subclinical plaque disruption or intracoronary thrombi [72].

Consequently, decisions regarding revascularization in T2MI should be individualized, taking into account the probability that the coronary lesion contributed to the initial supply–demand mismatch, as well as the patient’s symptom burden, left ventricular function, and procedural risk. Although the potential benefits of PCI in certain patients, such as those experiencing refractory symptoms or those with high-risk anatomy, are recognised, the prognostic benefit continues to be uncertain in the absence of randomised evidence. This underscores the importance of invasive coronary evaluation as a complementary strategy to refine diagnosis and facilitate tailored therapeutic decision-making.

To dispel any uncertainty, it should be noted that the prevalence of CAD in the course of T2MI, as described in the literature, varies depending on the diagnostic method used and the clinical definition adopted. While large clinical trials report the presence of any atherosclerotic plaque in 40–70% of patients with T2MI, advanced imaging studies assessing vascular anatomy (e.g., the DEMAND-MI study using CTA) indicate that some form of CAD (including subclinical atherosclerotic lesions) is present in as many as 68–92% of patients. Furthermore, approximately 26–30% of patients have angiographically significant stenotic disease, and previously undiagnosed CAD accounts for as many as two-thirds (approximately 66%) of cases.

## 7. Sex Differences

Sex and gender disparities are well-documented across a wide range of cardiovascular pathologies. While these variations are thoroughly characterized in T1MI, the influence of sex on the pathophysiology and clinical trajectory of T2MI remains poorly understood.

The reduced arterial caliber observed in females significantly limits coronary flow reserve, increasing the vulnerability of the female myocardium to ischemic injury during periods of increased metabolic demand. This susceptibility is further exacerbated by a higher prevalence of endothelial dysfunction in women [87,88]. These anatomical and physiological factors also contribute to an increased predisposition to SCAD, a non-atherosclerotic cause of T2MI. In SCAD, the expansion of a false lumen compresses the true lumen, resulting in severe impairment of myocardial perfusion [89]. While this condition can occur in any population, it demonstrates a pronounced female predominance, accounting for approximately 90% of cases [90].

The influence of sex hormones on vascular integrity is multifaceted and varies according to physiological context. While estrogen is traditionally recognized for its vasoprotective properties, its effects during pregnancy and the peripartum period can be paradoxical, compromising arterial wall strength by promoting smooth muscle relaxation and extensive extracellular matrix remodeling. This hormonal milieu increases susceptibility to vascular injury [91]. Subsequent withdrawal of this hormone, which is characteristic of the postpartum phase or perimenopausal transition, may precipitate dissection in vessels that have already become vulnerable [89,92]. In contrast, under stable physiological conditions, estrogens provide significant cardioprotective effects. They enhance endothelial function and regulate vascular tone via nitric oxide pathways [93], while also suppressing pro-inflammatory cytokines such as IL-6 and TNF-α [94]. The transition to menopause, characterized by a marked decline in estrogen levels, disrupts these homeostatic mechanisms. Such loss of hormonal protection is considered a principal factor in the increased incidence of T2MI among postmenopausal women, as it predisposes the microvasculature to dysfunction and vasoreactive episodes [91,95].

Testosterone exerts a complex, dual effect on the male vasculature. Endogenous concentrations of this hormone promote vasodilation in the coronary arteries, thereby improving myocardial oxygen delivery [96]. However, these same levels may impair vascular relaxation and exacerbate endothelial dysfunction when external stressors such as smoking or hypercholesterolemia are present [97]. Consequently, testosterone acts as a double-edged sword by modulating vascular tone in ways that may predispose individuals to T2MI under specific conditions. Additionally, reduced testosterone levels, which are frequently observed in metabolic syndrome and congestive heart failure, can compromise vascular reactivity and further exacerbate the supply–demand imbalance [98,99]. These hormonal and metabolic factors modify the underlying pathophysiology and increase the risk of T2MI [95,100].

Clinical data further corroborate these sex-specific physiological patterns. Rinaldi et al. [100] identified a distinct distribution of vasomotor dysfunction, with microvascular spasms occurring more frequently in women and epicardial coronary spasms occurring more commonly in men. Additionally, the influence of body mass index (BMI) on the risk of T2MI demonstrates notable sex specificity. Wang et al. [101] revealed that, among females, the risk of T2MI is significantly elevated in underweight (HR 4.06; 95% CI 1.41–11.44) and normal-weight groups (HR 3.47; 95% CI 1.64–7.36) compared to obese patients. In contrast, this association was not observed in men, indicating that obesity, which is a conventional cardiovascular risk factor, may affect the pathophysiology of T2MI differently between sexes.

The clinical presentation of T2MI is influenced by sex-specific differences in pain processing. While experimental data often show that women have higher pain sensitivity, they paradoxically experience more unrecognized infarctions [102].

This discrepancy may be partly due to the atypical symptoms in women, which are often masked by changes in pain thresholds during inflammatory states. Because T2MI is frequently triggered by systemic inflammation, these fluctuations can affect the perception of cardiac distress and contribute to diagnostic delays or misinterpretation of symptoms. Experimental models of LPS-induced inflammation demonstrate that immune activation selectively impairs endogenous pain inhibition (conditioned pain modulation) and increases pain sensitivity in females but not males, with pain sensitivity positively correlating with plasma IL-6 and IL-8 levels [103]. Therefore, optimal management in women requires the comprehensive exclusion of clinical mimics. Conditions such as Takotsubo Syndrome [104] and Myocarditis [105] and, in the peripartum context, peripartum cardiomyopathy (PPCM) [106] may present with similar features but necessitate distinct therapeutic approaches. Additionally, a systematic evaluation should assess for triggers more prevalent in women, such as severe anemia or thyroid dysfunction, as addressing these extra-cardiac causes is essential for resolving T2MI [107].

Epidemiological data indicate that women are generally older at presentation and exhibit more pronounced electrocardiographic signs of ischemia compared to men. Female patients demonstrate a higher prevalence of ST-segment depression and repolarization abnormalities, whereas men more frequently present with isolated T-wave inversion [108]. Lin et al. [108] reported that although coronary artery calcium (CAC) percentiles in patients with T2MI are elevated above the 50th percentile for both sexes, there is no significant difference between genders. In contrast, a substantial discrepancy is observed in plaque burden: women with T2MI consistently have lower total coronary plaque volumes than men across all age subgroups. These findings underscore a notable discordance between anatomical plaque morphology and the functional triggers of ischemia in women.

For any given degree of stenosis, women often demonstrate higher fractional flow reserve (FFR) values than men, which can misleadingly indicate preserved functional capacity despite significant arterial narrowing [109]. This anatomical–functional discordance is especially important in T2MI. Although favorable FFR values may suggest a low risk of epicardial ischemia, they frequently conceal a markedly reduced coronary flow reserve (CFR) [110]. This impairment, primarily resulting from coronary microvascular dysfunction (CMD), constitutes the main physiological substrate for T2MI in women. When clinical triggers such as tachycardia, severe anemia, or sepsis are present, the dysfunctional microvasculature is unable to increase blood flow, leading to a critical supply–demand mismatch even without obstructive coronary disease. Therefore, early CMR, ideally performed within two weeks of symptom onset, is crucial for diagnostic accuracy [111]. Using T1/T2 mapping and LGE, CMR enables the identification of non-ischemic mimics and frequently prompts the reclassification of cases initially diagnosed as ischemic. Bergamaschi et al. [112] demonstrated that the presence and extent of LGE independently predict adverse cardiac events at approximately three years of follow-up. Thus, CMR functions not only as a diagnostic modality but also as a fundamental component of personalized care.

Evidence demonstrates a persistent gender disparity in the clinical management of T2MI, reflecting patterns also observed in T1MI, where women are less likely to undergo revascularization and receive comprehensive secondary prevention. Female patients diagnosed with T2MI are less likely to receive optimal medical therapy (OMT) or to be subjected to thorough diagnostic evaluations compared to male patients [103]. This therapeutic gap is frequently exacerbated by a fundamental diagnostic bias. Specifically, the absence of obstructive CAD on initial imaging often results in clinicians underestimating the severity of underlying ischemia. As previously discussed, women frequently exhibit a substantial atherosclerotic substrate, as indicated by elevated CAC scores. However, this plaque burden is often non-obstructive and therefore remains undetected by conventional angiographic assessment [113].

A comprehensive analysis of 6485 patients by Kimenai et al. [103] identified significant sex-based differences in the diagnostic and therapeutic trajectories in T2MI. Although the rates of invasive investigation were similar for both sexes, women were twice as likely as men to have non-obstructive coronary arteries (33% versus 66%), resulting in a lower coronary revascularisation rate (9% vs. 16%). This angiographic profile was directly associated with a lower likelihood of being prescribed statins at discharge, even after adjusting for comorbidities. However, sex was not identified as an independent predictor of statin prescription (OR: 0.84; 95% CI: 0.67–1.05).

The clinical significance of T2MI is further underscored by its profound impact on long-term prognosis. According to Kimenai et al. [103], patients with T2MI have a higher mortality rate than those with T1MI, with all-cause mortality observed in 50% of men and 45% of women, compared to 22% and 29% in T1MI, respectively. Both all-cause mortality and major adverse cardiovascular events (MACE), which affect over half of these patients, were more prevalent in men than in women. Although T2MI presents a significant risk to both sexes, women demonstrated a survival advantage. After adjustment for comorbidities and pharmacological treatment, female patients had a 19% lower risk of death compared to male patients (HR 0.81; 95% CI 0.74–0.90). These findings indicate that, despite therapeutic disparities, the pathophysiological mechanisms underlying T2MI may confer a greater mortality risk in men.

Further insights are provided by Ariss et al. [114], whose analysis of over 250,000 hospitalizations revealed distinct comorbidity profiles between the sexes. Female patients were more likely to be older and presented with a higher prevalence of non-cardiovascular triggers, including deficiency anemias, hypothyroidism, and chronic pulmonary disease. In contrast, males exhibited a higher burden of established cardiovascular disease, such as prior MI, dyslipidemia, and atrial fibrillation. The study further identified sex-specific predictors of in-hospital mortality. While age, coagulopathy and liver disease were found to be risk factors for both sexes, heart failure, chronic pulmonary disease and peripheral vascular disease were identified as significant predictors of female mortality, but not male mortality. Furthermore, females had a lower risk of in-hospital death compared to men (aOR 0.92; 95% CI 0.88–0.96), a finding that aligns with European cohorts (such as SWEEDHEART [79] or APACE [115]) and emphasizes the need to tailor risk stratification based on sex-specific comorbidity patterns.

Diagnosing T2MI in women is particularly difficult due to sex-related differences in biomarker kinetics and symptom presentation. While serial high-sensitivity cardiac troponin I (hs-cTnI) measurements demonstrate that sensitivities for T2MI improve substantially over time—reaching >95% within 2 h—females tend to present with lower troponin levels at the time of admission [116,117]. This disparity, likely reflecting smaller infarct sizes or times of presentation in women, necessitates the application of sex-specific 99th percentile URLs, such as 16 ng/L for females versus 34 ng/L for males proposed by Sandoval et al. [47], to ensure diagnostic accuracy.

The challenges in comparing prognosis and therapeutic strategies in T2MI underscore the inherent complexity and heterogeneity of this condition. Given the diverse pathophysiological mechanisms, patient characteristics—including sex—play a pivotal role in determining clinical outcomes. These findings emphasize the urgent need for further research into sex-specific factors to facilitate tailored management and ultimately improve long-term outcomes for both men and women.

## 8. Conclusions

The clinical management of T2MI poses significant diagnostic and therapeutic challenges, largely due to its heterogeneous pathophysiology and the absence of standardized clinical guidelines. Non-ischemic myocardial injury frequently confounds the diagnosis of T2MI, which is further intensified by the subjective nature of symptoms and persistent academic debates regarding its classification.

The diagnostic process is additionally complicated by highly variable and often atypical clinical presentations, which impede the identification of a single, definitive etiology. As a result, rigorous differential diagnosis is essential for accurate identification and the implementation of individualized treatment strategies. Although traditional diagnostic tools, such as ECG, laboratory markers such as CRP, hs-cTn, NT-proBNP, and more, are valuable for initial screening, they frequently lack the specificity required to distinguish T2MI from T1MI when used alone. Therefore, a multidisciplinary and multi-level approach is critical for improving clinical outcomes. The integration of novel biomarkers, standardized diagnostic protocols, and systematic exclusion of T1MI are necessary steps toward achieving diagnostic precision. In addition, advanced non-invasive imaging modalities, including CCTA and CMR, are essential for characterizing ischemic patterns and providing comprehensive structural information. Given the uncertainty regarding the benefits of routine invasive strategies, therapeutic decisions should be individualized, carefully balancing ischemic risk with patient-specific vulnerabilities. Ultimately, prognoses and therapeutic outcomes highlight the inherent complexity of T2MI. Patient characteristics, such as biological sex and comorbidities, significantly influence clinical trajectories. Despite recent significant advances, further evidence-based research is necessary to address existing knowledge gaps and to refine diagnostic and therapeutic strategies for this heterogeneous patient population.

## Data Availability

No new data were created or analyzed in this study. Data sharing is not applicable to this article.

## Abbreviations

The following abbreviations are used in this manuscript:

AMI	Acute Myocardial Infarction
aOR	Adjusted Odds Ratio
APACE	Advantageous Predictors of Acute Coronary Syndromes Evaluation
AUC	Area Under the Curve
BACC	Biomarkers in Acute Cardiovascular Care
BMI	Body Mass Index
CAC	Coronary Artery Calcium
CAD	Coronary Artery Disease
CCTA	Coronary Computed Tomography Angiography
CFR	Coronary Flow Reserve
CHOPIN	Copeptin Helps in the early detection Of Patients with acute myocardial Infarction
CI	Confidence Interval
CK-MB	Creatine Kinase Myocardial Band
CMD	Coronary Microvascular Dysfunction
CMR	Cardiac Magnetic Resonance
CRP	C-Reactive Protein
CT-proET-1	C-terminal pro-Endothelin-1
cTnI	Cardiac Troponin I
cTnT	Cardiac Troponin T
ECG	Electrocardiogram/Electrocardiography
EF	Ejection Fraction
ESC	European Society of Cardiology
FFR	Fractional Flow Reserve
FFR-CT	CT-derived Fractional Flow Reserve
Gal-3	Galectin-3
GLS	Global Longitudinal Strain
HR	Hazard Ratio
hs-cTn	High-Sensitivity Cardiac Troponin
hs-cTnI	High-Sensitivity Cardiac Troponin I
IL-6	Interleukin-6
IL-8	Interleukin-8
IVUS	Intravascular Ultrasound
LGE	Late Gadolinium Enhancement
LPS	Lipopolysaccharide
MACEs	Major Adverse Cardiovascular Events
MI	Myocardial Infarction
MINOCAs	Myocardial Infarction with Non-Obstructive Coronary Arteries
MR-proADM	Mid-Regional pro-Adrenomedullin
MR-proANP	Mid-Regional pro-Atrial Natriuretic Peptide
NSTEMI	Non-ST-Elevation Myocardial Infarction
NT-proBNP	N-terminal pro-B-type Natriuretic Peptide
OCT	Optical Coherence Tomography
OMT	Optimal Medical Therapy
OR	Odds Ratio
P	P-value
PCI	Percutaneous Coronary Intervention
PPCM	Peripartum Cardiomyopathy
RWMAs	Regional Wall Motion Abnormalities
SCAD	Spontaneous Coronary Artery Dissection
STEMI	ST-Elevation Myocardial Infarction
SWEDEHEART	Swedish Web-system for Enhancement and Development of Evidence-Based Care in Heart disease Evaluated According to Recommended Therapies
T1MI	Type 1 Myocardial Infarction
T2MI	Type 2 Myocardial Infarction
TNF-α	Tumor Necrosis Factor-alpha
TTE	Transthoracic Echocardiography
URL	Upper Reference Limit
